# Supplementary figures and images for: Endogenous Pain Modulation in Response to a Single Session of Percutaneous Electrolysis in Healthy Population: A Double-Blinded Randomized Clinical Trial
Source: J Clin Med. 2022 May 20;11(10):2889. doi: 10.3390/jcm11102889 (PMC9143044; doi:10.3390/jcm11102889)

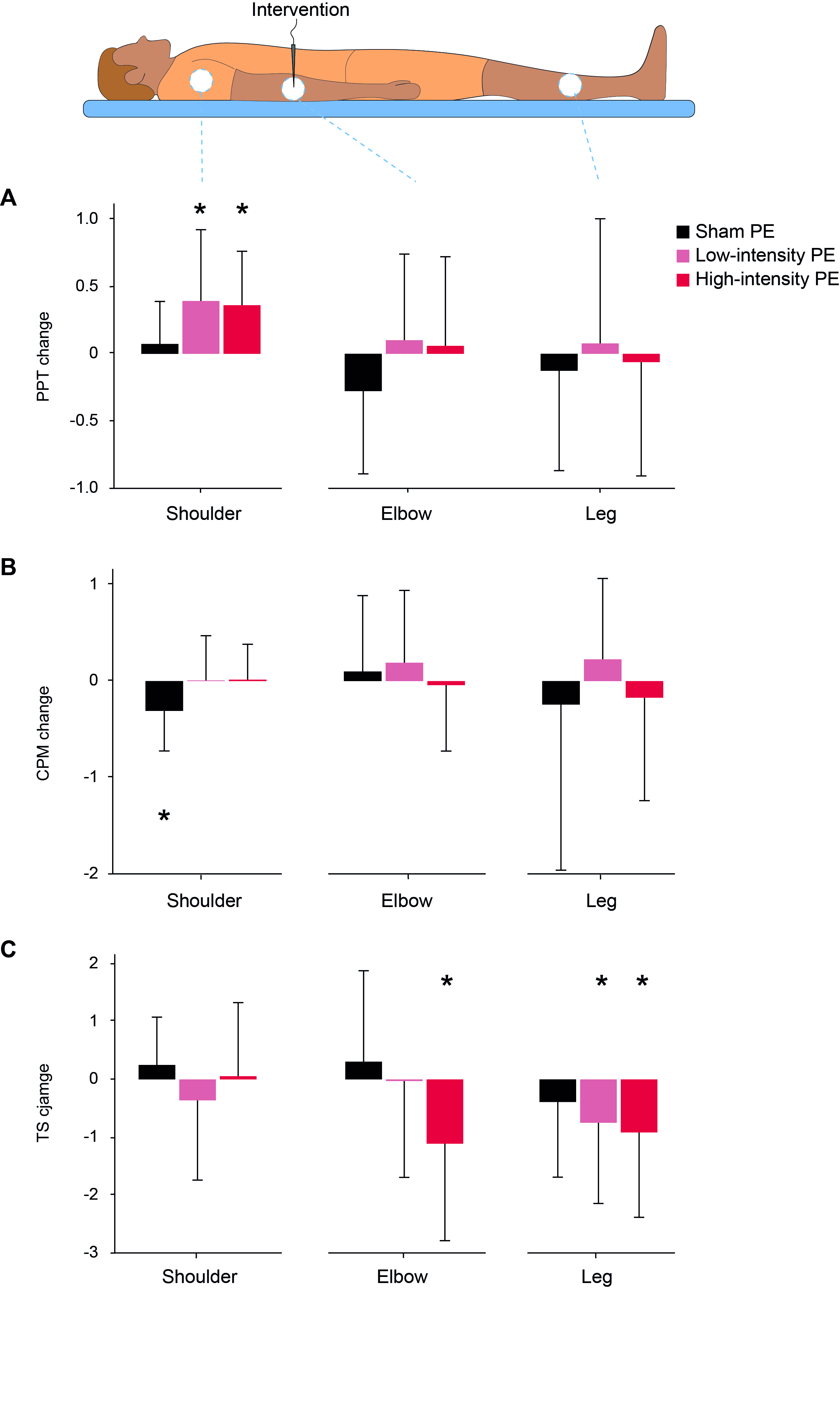

Supplement: Supplementary file 1 [file jcm-11-02889-s001.zip › jcm-1697039-supplementary.jpg]
